# Psychiatric Stigma in Treatment-Seeking Adults with Personality Problems: Evidence from a Sample of 214 Patients

**DOI:** 10.3389/fpsyt.2015.00101

**Published:** 2015-07-08

**Authors:** Kirsten Catthoor, Didier Schrijvers, Joost Hutsebaut, Dineke Feenstra, Bernard Sabbe

**Affiliations:** ^1^Psychiatrisch Ziekenhuis Stuivenberg, Ziekenhuis Netwerk Antwerpen, Antwerp, Belgium; ^2^Collaborative Antwerp Psychiatric Research Institute (CAPRI), Antwerp University, Antwerp, Belgium; ^3^Universitaire Dienst Psychiatrie, Psychiatrisch Ziekenhuis Sint-Norbertushuis, Duffel, Belgium; ^4^Viersprong Institute for Studies on Personality Disorders (VISPD), Halsteren, Netherlands

**Keywords:** stigma, adult patients, personality disorders, psychotherapy, mental health burden

## Abstract

Stigmatization is a major burden in adult psychiatric patients with Axis-I diagnoses, as shown consistently in most studies. Significantly fewer studies on the emergence of psychiatric stigma in adult patients with personality disorders (PDs) exist, although the resulting evidence is conclusive. Some authors consider patients with PDs at risk for severe stigmatization because of intense difficulties during interpersonal contact, even in a psychotherapeutic relationship. The aim of this study was primarily the assessment of pre-existing stigma in patients referred for intensive treatment for PDs. The study enrolled 214 patients admitted to the adult department of a highly specialized mental health care institute offering psychotherapy for patients with severe and complex personality pathology. All patients underwent a standard assessment with self-report questionnaires and a semi-structured interview to measure Axis II PDs. The stigma consciousness questionnaire and the perceived devaluation-discrimination questionnaire, both validated instruments, were used to measure perceived and actual experiences of stigma. Independent sample *t*-tests were used to investigate differences in the mean total stigma scores for patients both with and without a PD. One-way ANOVAs were performed to assess the differences between having a borderline PD, another PD, or no PD diagnosis. Multiple regression main effect analyses were conducted in order to explore the impact of the different PD diagnosis on the level of stigma. The mean scores across all patient groups were consistent with rather low stigma. No differences were found for patients with or without a PD diagnosis. Level of stigma in general was not associated with an accumulating number of PDs. Given the remarkable results, we would strongly recommend further investigations in the field to better understand the phenomenon of stigma in all its aspects.

## Introduction

A stigma is defined as a discrediting and disgracing mark that usually leads to negative behavior in its bearer (1–10). Various approaches have been used to conceptualize psychiatric stigma (1–6). The definition and conceptualization of psychiatric stigma are still in full evolution. Aspects of devaluation, discrimination, decreased self-esteem, self-restricted behavior, and dysfunctional coping are almost always mentioned (1–4).

The conclusion that stigmatization is experienced as a major burden for adult psychiatric patients with Axis-I diagnoses has been shown consistently in most studies (7–14). It is also clear that self-stigma is common; consistently related to social anxiety and depression; and correlated with lower self-esteem, lower therapeutic adherence, and negative quality of life (8, 10, 12, 13). There are significantly fewer studies on the emergence of psychiatric stigma in adult patients with personality disorders (PD), although the existing evidence is conclusive (11, 15). Rüsch et al. found that borderline patients are vulnerable to higher stigmatization because of the severity of their conditions, common interpersonal difficulties, and visible devaluating signs, such as self-mutilation scars (15). Stigma in borderline patients is associated with lower self-esteem and other signs of poorer psychological well being (15). In a qualitative study, Dinos et al. showed that patients with PD who did not experience overt discrimination, were affected by patronizing attitudes and stigma anyway (11).

Aviram (16) considers sufferers of PD at risk for severe stigmatization because of the possible intense difficulties triggered by and experienced during interpersonal contact. Several other studies (17, 18) emphasize that the belief in a weak or nervous personality as the cause of mental disorders, is consistently associated with more stigma. Other authors (19) state that increasing the public understanding of the biological substrates of mental illness does not result in a better social acceptance of psychiatric patients. Interpersonal difficulties between patients and therapists might also perpetuate negative perceptions in mental health care workers (16). The widespread idea that the behavior of patients with PD is deliberate and within their control and the misconception of their rejection of offered help enhance the chance for pejorative, judgmental, and rejecting attitudes. Especially in the treatment of borderline patients with frightening symptoms including self-harm and suicidal attempts, distancing, and self-protection of the therapist can lead to a self-fulfilling prophecy of more stigmatization and prejudice. Self-esteem and personal empowerment are basic features in the treatment of PDs and are closely related to basic assumptions in stigma theories, as mentioned above. Stigma research (20, 21) also has revealed that self-esteem can mediate between internalized stigma and hope. Promoting personal empowerment might challenge stigma in a similar way.

A recent study showed that treatment-seeking adolescents with PD experience more stigma than treatment-seeking adolescents with other severe and treatment refractory psychiatric disorders (22). Borderline PD was shown to be the strongest predictor of experiences of stigma in this group of treatment-seeking personality disordered adolescents, when controlled for other types of personality pathology. Adolescents with a combination of different kinds of personality diagnoses tend to experience the highest levels of stigma. The authors suggest that therapists should be aware of psychiatric stigma when treating these sub-groups of adolescents with personality problems and discuss its existence with their patients and families. Psycho-education about the nature and etiology of PD and treatment prognosis could be helpful interventions too.

Given the evidence for higher stigma in treatment-seeking adolescents with PDs, it is legitimate to hypothesize that stigmatization in treatment-seeking adults with PDs will be more pronounced than stigmatization in treatment-seeking adults without PDs. The aim of this study was to assess whether there is a difference in levels of stigmatization experienced by treatment-seeking adults diagnosed with PD, in comparison with other treatment-seeking adults.

## Materials and Methods

This study was part of the Study on Cost-Effectiveness of Personality disorder Treatment (SCEPTRE)-trial, a multi-center study conducted with the purpose of providing data for economic evaluation of various psychotherapeutic treatments for adults with PDs. The study was registered in the Dutch trial register (ISRCTN: 73817429) and received approval of the Medical Ethics Committee of the Erasmus University Medical Centre (Rotterdam, Netherlands). All included patients gave informed consent (23). Inclusion criteria for the study were patients with long existing psychiatric symptoms suggesting personality pathology. Comorbid Axis-I disorders were allowed. Exclusion criteria were patients with psychotic disorders, severe behavioral disturbances, organic cerebral impairment, and mental retardation. The stigma-study enrolled 216 patients from SCEPTRE in De Viersprong. All participating SCEPTRE-patients were asked to cooperate in the stigma-study, but they could easily refuse without giving an explanation. All patients included in the stigma-trial explicitly provided oral informed consent to complete the stigma questionnaires. De Viersprong is a highly specialized mental health care institute offering outpatient, day hospital, and inpatient psychotherapy for adolescents and adults with severe and complex personality pathology. The majority of patients have a long history of severe psychiatric symptoms and problems whereby previous treatments were often unsuccessful. De Viersprong aims to achieve sustainable patient recovery with innovative and proven cost-effective treatments. All patients undergo a standard assessment as part of the intake procedure, to assess the severity of personality problems and to determine their eligibility for psychotherapeutic treatment. The intake procedure includes self-report questionnaires to measure psychopathology, personality, functional impairments, and treatment history, and a semi-structured interview to measure Axis II PDs. Interviewers were masters-level psychologists who were thoroughly trained and received monthly booster sessions to avoid drifting from the interviewer guidelines. Two patients did not complete the assessment battery as part of the formal admission procedure, which left 214 patients for the current sample.

### Measures

#### Diagnostic Interview

The structured interview for the DSM-IV personality (SIDP-IV) (24, 25) was used to measure PDs. This interview covers the 11 formal DSM-IV-TR Axis II PD diagnoses, including personality disorder not otherwise specified (PDNOS), two appendix diagnoses (depressive and negativistic PD), and the DSM-III-R self-defeating PD. Inter-rater reliability was computed by videotaped interviews, with percentages of agreement ranging from 84% (avoidant PD) to 100% (schizoid PD).

#### Questionnaires Measuring Stigma

The stigma consciousness questionnaire (SCQ) (26) is used to measure perceived and actual experiences of stereotypes in specific target groups. It is a validated instrument consisting of 10 items scored on a Likert scale ranging from 1 to 6. The lower the score, the higher the level of stigma consciousness. A Dutch translation of the questionnaire was used in this study, and it was based on a forward and backward translation.

The perceived devaluation-discrimination questionnaire (DDQ) (27) is used to measure the individual perception of how “most other people” view individuals with mental illness. The scale is widely used in stigma research (28), has excellent psychometric properties, and predicts deterioration in self-esteem. It consists of 12 items rated on a Likert scale with a range from 1 (highest awareness) to 6 (lowest awareness). The results of the scale are obtained by summing the items and dividing by 12. A Dutch translation of the questionnaire was used in this study based on a forward and backward translation. Because of an error in the translation process, one item (question 7) was left out of the version used in our analyses. There are no data available for the use of this questionnaire in personality disordered patient groups. A cohort consisting of adult patients from different diagnostic categories (schizophrenia, major depressive disorder, and schizophrenia-like psychotic disorders) all showed mean scores on the DDQ that were significantly different from the midpoint with *p* < 0.001 (29).

### Statistical procedures

Independent sample *t*-tests were used to investigate differences in the mean total scores for the questionnaires measuring stigma for patients both with and without a PD. One-way ANOVAs were performed to assess the differences between having a borderline PD, another PD, or no PD diagnosis. Multiple regression main effect analyses were conducted in order to explore the impact of the different PD diagnosis on the level of stigma. Age and gender were also entered in the regression models. To investigate the relationship between accumulative personality pathology and the level of stigma, we observed the trend in stigma scores (for both the SCQ and the DDQ) with an increasing number of PDs.

## Results

### Participants

Of the 214 patients admitted to de Viersprong, 133 (62.1%) were female and 81 were male (37.9%). Participants were 19–67 years of age with a mean age of 33.9 (SD = 10.03). One hundred twenty-seven patients were formally diagnosed with a PD, from whom 54 had 1 PD, 34 had 2 PDs, and 39 had 3 or more PD. Seventy-seven patients were not diagnosed with a PD, although all patients in this group showed at least 7 personality traits on SIDP-IV. PDNOS was most frequently diagnosed (38.3%), followed by avoidant PD (22%), depressive PD (19.6%), and borderline PD (13.6%). Schizotypal PD and negativistic PD were the least frequently diagnosed disorders (with both at 0.5%).

The mean SCQ score for the total group of patients was 4.38 (SD = 1.07). The average DDQ score was 4.60 (SD = 0.77). The mean scores across all patient groups were significantly higher than the midpoint of the scale, which was consistent with rather low stigma consequences.

### Personality disorder versus no personality disorder

As shown in Table 1, no differences were found for patients with or without a PD diagnosis.

**Table 1:** **Mean stigma scores of patients both with and without a PD (*n* = 204–209)^a^**.

Questionnaire	Patients with PD (*n* = 127–131)^a^	Patients without PD (*n* = 77–78)^a^	*t*	*p*	*d*
	*M* (SD)	*M* (SD)	
Stigma consciousness questionnaire (SCQ)	4.36 (1.00) (*n* = 131)	4.41 (1.19) (*n* = 78)	0.280	0.779	0.05
Devaluation-discrimination questionnaire (DDQ)	4.58 (0.76) (*n* = 127)	4.64 (0.79) (*n* = 77)	0.513	0.608	0.08

*PD, personality disorder*.

*^a^*N* varies due to missing values*.

### Borderline personality disorder versus other personality disorder versus no personality disorder

When assessing the differences on both stigma questionnaires for having a borderline PD (*n* = 27–29, for DDQ and SDQ, respectively), another PD (*n* = 100–102, for DDQ and SDQ, respectively) or no PD (*n* = 77–78, for DDQ and SDQ, respectively), no significant differences were found between the three groups [SCQ: *F*(2,206) = 0.045, *p* > 0.05; and DDQ: *F*(2,201) = 0.191, *p* > 0.05].

### Stigma by type of personality disorder

Stigma consciousness questionnaire and DDQ values for the different PD diagnoses are presented in Tables 2 and 3. The results shown in Table 2 suggest that patients with schizoid PD experience the highest level of stigma, with a SCQ score of 3.45. No PD, however, could significantly predict the level of stigma as measured by the SCQ [*F*(16,192) = 0.780, *p* > 0.05]. As for the results obtained using the DDQ [*F*(16,187) = 0.983, *p* > 0.05], paranoid PD significantly predicted higher levels of experienced stigma.

**Table 2:** **Mean SCQ scores for DSM-IV Axis II personality disorders (*n* = 209)**.

	*N* (%)	SCQ *M* (SD)	Analysis			
			*B* (SE)	CI		β
				Lower bound	Upper bound	
Age			0.009 (0.01)	−0.007	0.025	0.084
Gender			−0.212 (0.17)	−0.549	0.125	−0.096
Paranoid PD	4 (1.9)	4.03 (0.38)	−0.337 (0.56)	−1.446	0.772	−0.043
Schizoid PD	4 (1.9)	3.45 (1.25)	−0.949 (0.64)	−2.215	0.317	−0.122
Schizotypal PD	1 (0.05)	3.8 (–)	0.011 (1.27)	−2.487	2.509	0.001
Antisocial PD	2 (1.0)	4.65 (1.20)	0.118 (0.78)	−1.429	1.664	0.011
Borderline PD	29 (13.9)	4.34 (0.83)	−0.058 (0.23)	−0.512	0.396	−0.019
Histrionic PD	3 (1.4)	3.87 (0.80)	−0.676 (0.66)	−1.969	0.617	−0.075
Narcissistic PD	6 (2.9)	4.23 (0.78)	−0.340 (0.47)	−1.259	0.579	−0.053
Avoidant PD	46 (22.0)	4.37 (1.03)	0.027 (0.21)	−0.381	0.435	0.010
Dependent PD	17 (8.1)	4.46 (1.07)	0.134 (0.31)	−0.471	0.739	0.034
Obsessive-compulsive PD	21 (10.0)	4.21 (1.03)	−0.073 (0.26)	−0.581	0.436	−0.020
Self-defeating PD	11 (5.3)	4.79 (1.00)	0.545 (0.37)	−0.187	1.276	0.114
Depressive PD	42 (20.1)	4.27 (1.03)	−0.238 (0.22)	−0.674	0.198	−0.089
Negativistic PD	1 (0.05)	4.00 (–)	−0.610 (1.12)	−2.810	1.590	−0.039
Personality disorder NOS	80 (38.3)	4.45 (1.00)	0.195 (0.17)	−0.131	0.521	0.089
Any PD	131 (62.7)	4.36 (1.19)			
No PD	78 (37.3)	4.41 (1.00)			

*SCQ, stigma consciousness questionnaire; PD, personality disorder; NOS, not otherwise specified; CI, confidence interval (95%)*.

*The sum of the number of patients in the different diagnostic groups is higher than the total number of patients because patients can have more than one personality disorder*.

*p < 0.05; **p < 0.01; ***p ≤ 0.001; *R* = 0.25.

**Table 3:** **Mean DDQ scores for DSM-IV Axis II personality disorders (*n* = 204)**.

	*N* (%)	SCQ *M* (SD)	Analysis			
			*B* (SE)	CI		β
				Lower bound	Upper bound	
Age			−0.000 (0.00)	−0.012	0.012	0.000
Gender			−0.038 (0.13)	−0.283	0.208	−0.023
Paranoid PD	4 (2.0)	3.58 (0.50)	−1.010 (0.40)	−1.803	−0.216	−0.182*
Schizoid PD	4 (2.0)	4.53 (0.73)	−0.102 (0.46)	−1.008	0.805	−0.018
Schizotypal PD	1 (0.5)	4.3 (–)	−0.316 (0.91)	−2.104	1.471	−0.029
Antisocial PD	2 (1.0)	4.8 (0.71)	0.046 (0.56)	−1.062	1.153	0.006
Borderline PD	27 (13.2)	4.63 (0.78)	0.110 (0.17)	−0.226	0.447	0.049
Histrionic PD	3 (1.5)	3.97 (1.0)	−0.620 (0.47)	−1.545	0.305	−0.097
Narcissistic PD	6 (2.9)	4.87 (0.84)	0.209 (0.33)	−0.450	0.867	0.046
Avoidant PD	43 (21.1)	4.55 (0.74)	−0.081 (0.152)	−0.382	0.219	−0.043
Dependent PD	17 (8.3)	4.34 (0.81)	−0.271 (0.22)	−0.706	0.165	−0.097
Obsessive-compulsive PD	21 (10.3)	4.44 (0.62)	−0.105 (0.19)	−0.470	0.259	−0.041
Self-defeating PD	11 (5.4)	4.5 (0.95)	0.046 (0.27)	−0.479	0.571	0.014
Depressive PD	29 (19.1)	4.65 (0.74)	0.152 (0.16)	−0.172	0.475	0.077
Negativistic PD	1 (0.5)	3.70 (–)	−1.169 (0.80)	−2.745	0.407	−0.106
Personality disorder NOS	78 (38.2)	4.66 (0.74)	0.091 (0.12)	−0.144	0.326	0.057
Any PD	127 (62.3)	4.58 (0.76)			
No PD	77 (37.7)	4.64 (0.79)			

*DDQ, devaluation-discrimination questionnaire; PD, personality disorder; NOS, not otherwise specified; CI, confidence interval (95%)*.

*The sum of the number of patients in the different diagnostic groups is higher than the total number of patients because patients can have more than one personality disorder*.

*p < 0.05; **p < 0.01; ***p ≤ 0.001; *R* = 0.28.

### Stigma by accumulative personality pathology

Table 4 shows that the experienced level of stigma does not increase with an accumulating total number of PDs. There was no effect of the total number of PDs on mean total stigma scores. Although the patient groups with five or six PDs are small, it is clear that there is no single tendency toward more stigma associated with more PDs.

**Table 4:** **SCQ (*n* = 209) and DDQ (*n* = 204) scores by the total number of personality disorders (*n* = 204–209)^a^**.

Number of PDs	SCQ		DDQ	
	*M*	SD	*M*	SD
No PD (*n* = 78/77)^a^	4.41	1.19	4.64	0.79
1 PD (*n* = 55/54)^a^	4.31	1.00	4.50	0.79
2 PDs (*n* = 35/34)^a^	4.51	0.98	4.79	0.80
3 PDs (*n* = 28/27)^a^	4.31	1.10	4.53	0.63
4 PDs (*n* = 9/8)^a^	4.46	1.02	4.78	0.62
5 PDs (*n* = 2)	3.55	0.21	4.65	0.64
6 PDs (*n* = 2)	4.40	0.42	3.20	0.00

*SCQ, stigma consciousness questionnaire; DDQ, devaluation-discrimination questionnaire; PD, personality disorder*.

*^a^*N* varies due to missing values*.

## Discussion

This study aimed to investigate basic levels of stigmatization in patients with PDs referred for intensive psychotherapeutic treatment. Given the consistent emphasis in the literature on substantial stigma in patients with PDs (11), the scientific evidence for higher stigma in women with borderline PD compared to those with social phobia (15), and the significant differences in stigma between personality disordered adolescents and otherwise psychiatrically disturbed adolescents (23), we expected remarkable differences in stigma between patients with and without PDs.

Results of our study were not in line with these expectations: (1) mean total stigma scores were generally low in all patient groups; (2) there was no impact of a diagnosis of PD on the experienced stigma; (3) there was no effect of the number of PDs on experienced stigma; and (4) there were no differences found between different types of PDs. Only paranoid PD showed significantly higher stigma scores on the DDQ, which can be understood as inherent to this type of disorder: a basic lack of trust in human interactions. The main result of the study is that there are no differences in stigma between treatment-seeking adults with or without PD. Similar studies with patient cohorts consisting of different diagnostic categories (schizophrenia, major depressive disorder, schizophrenia-like psychotic disorders and adolescents with PD) showed marked higher stigma in all patient groups (23, 29).

Several hypothetical explanations could explain our results. First, there was bias in the features of the participants. All participating patients had seven or more personality traits. Patients from the non-personality disturbed group actually showed at least tendency toward personality pathology. Although there were clear differences in both groups concerning the classification of a categorical personality diagnosis, we must consider overlap in personality characteristics. We can assume that our sample of patients was rather homogeneous regarding maladaptive personality traits. Besides, all participants were all able to perform a complex, demanding, and sometimes frustrating intake procedure, indicating that their personality organization was stable enough to bear this task. More severely disturbed patients are not admitted to the intake procedure, such as borderline patients with documented serious self-harmful behavior. Second, an intense intake procedure can give hope and improve self-esteem; patients may be convinced that a careful approach and adherence to psychotherapeutic treatment finally will lead to a better chance for recovery. This hope and improved self-esteem can provide a buffer for feelings of stigmatization. Third, the participants were not yet involved in an intensive psychotherapeutic relationship with a mental health professional, which is considered to be a serious risk factor by several authors (16).

Other possible explanatory hypotheses are the nature of PDs, with an overload of internalized problems that overflow external attributions such as stigma. In addition, patients referred for intensive psychotherapeutic treatment might not identify themselves as psychiatric patients, who make the method and questionnaires inappropriate methods for assessing this type of information.

Limitations of the study were a bias in patient sample: personality disordered patients with severe behavioral disturbances were excluded. Some patients from the SPECTRE-study refused to participate in the stigma-study. It should be taken into account that patients from a stigma-sensible environment could refuse to participate in this study. We also must consider that some sub-groups consist of only a very limited number of patients, which therefore limit the interpretation. Another limitation in this study is the lack of assessed Axis-I disorders, what makes it difficult to describe the group of patients without PD. The current study’s strengths are the large study cohort.

To the best of our knowledge, this is the first study to measure stigma in a large sample of patients referred for intensive treatment of PDs. Given the remarkable results, we would strongly recommend further investigations in the field, both before and after the psychotherapeutic treatment, comparing other diagnostic groups and using other questionnaires, to better understand the phenomenon.

## Conflict of Interest Statement

The authors declare that the research was conducted in the absence of any commercial or financial relationships that could be construed as a potential conflict of interest.
